# Transactions of the American Dental Association

**Published:** 1890-12

**Authors:** 


TRANSACTIONS OF THE AMERICAN DENTAL ASSOCIATION at the 30th Annual Session, held at Excelsior Springs, Mo., commencing August 5th, 1890. Chicago, H. D. Justi: The Dental Review Co., 1890, Publishers. This is a valuable volume of proceedings, and includes instructive articles read before the Association with the discussions on each one.

